# The Expanding Constellation of Histone Post-Translational Modifications in the Epigenetic Landscape

**DOI:** 10.3390/genes12101596

**Published:** 2021-10-10

**Authors:** Vincenzo Cavalieri

**Affiliations:** Department of Biological, Chemical and Pharmaceutical Sciences and Technologies, University of Palermo, 90128 Palermo, Italy; vincenzo.cavalieri@unipa.it

**Keywords:** histone post-translational modifications, chromatin, nucleosome, epigenetics

## Abstract

The emergence of a nucleosome-based chromatin structure accompanied the evolutionary transition from prokaryotes to eukaryotes. In this scenario, histones became the heart of the complex and precisely timed coordination between chromatin architecture and functions during adaptive responses to environmental influence by means of epigenetic mechanisms. Notably, such an epigenetic machinery involves an overwhelming number of post-translational modifications at multiple residues of core and linker histones. This review aims to comprehensively describe old and recent evidence in this exciting field of research. In particular, histone post-translational modification establishing/removal mechanisms, their genomic locations and implication in nucleosome dynamics and chromatin-based processes, as well as their harmonious combination and interdependence will be discussed.

## 1. Introduction

Chromatin is a polymeric nucleoprotein complex composed primarily of DNA closely associating with positively charged histone proteins [1]. The basic structural repeating unit is the nucleosome core particle, consisting of 147 base pairs of DNA wrapped nearly twice in a left-handed toroidal supercoil around a core histone octamer [2]. The latter exhibits a tripartite organization, which comprises a tetramer containing two copies of H3 and H4, coupled to two H2A-H2B dimers [3]. Each core histone contains a globular histone fold domain, which mediates heterodimeric interactions, and two amino- and carboxy-terminal unstructured extensions protruding from the surface of the nucleosome [4]. These flexible domains, commonly known as the histone tails, provide additional stabilization effects through their contacts with the nucleosomal DNA backbone and acidic patch of adjacent nucleosomes [5]. As an additional hierarchical level of chromatin compaction, core particles are further stabilized by the binding of histone H1 at the two linker DNA arms entering and exiting on the surface of each octamer [6,7]. Molecular processes establishing such a histone stoichiometry determines the basic chromatin fiber structure [8,9].

**Figure 1:**
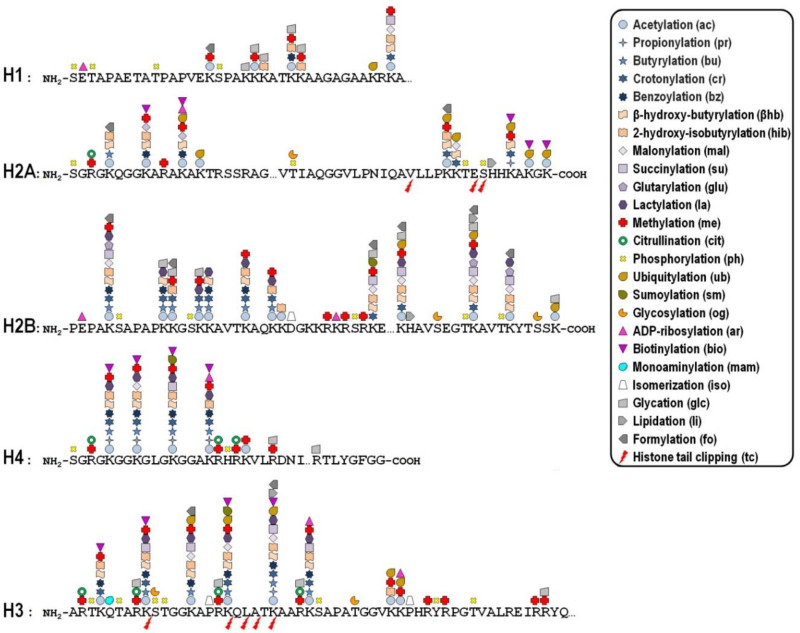
Diagrammatic representation showing the distribution of post-translational modifications within the N- and C-terminal portions of the five canonical histones. The first amino acid methionine was omitted from the sequences. Key for each histone PTM is included in diagram. Reference sequences of histone proteins were retrieved from the NCBI protein database (https://www.ncbi.nlm.nih.gov/protein?cmd=retrieve; accessed on 2 October 2021) using the following accession numbers: NP_005313.1 (H1), NP_003504.2 (H2A), NP_066406.1 (H2B), NP_003520.1 (H3), and NP_003529.1 (H4).

Key mechanisms contributing to the dynamic regulation of the degree of chromatin compaction to allow the harmonious utilization of the genetic information include covalent post-translational modifications (PTMs) of histones [10,11]. In this regard, histone tails represent preferential, but not exclusive, targets of a myriad of PTMs that correlate with changes in gene activity. In fact, the recent improvement of the sensitivity of mass spectrometry-based methods allowed the discovery of more than two hundred histone modification sites so far (Figure 1), although no functional significance has been assigned to the majority of them [12,13]. These modifications are imposed, and can be reversed as well, by an assortment of histone-modifying enzymes, so that they represent a major mechanism of cellular adaptation to the varying environmental conditions [14,15,16]. Broadly speaking, the presence of histone PTMs alters the stereochemical environment of the nucleosomes and their binding affinity for regulatory complexes that can be recruited or turned away from chromatin, thereby eliciting functional outcomes.

Whether a given histone PTM correlates with gene expression in a positive or negative fashion most likely depends on its genomic location. To make matters more complicated, it is now becoming clear that the combinatorial nature of histone PTMs implies both the coalescence of multiple modifications on the same nucleosome and the possibility of functional promiscuity induced by synergistic or antagonistic cross-talk among them. The simplest example of such a cross-talk is the direct competition, on the same histone residue, among alternative PTMs associated with functionally opposite transcriptional readouts. In many other cases, one modification promotes/inhibits the generation/removal of another modification “in cis”, viz within a distinct site in the same histone, or “in trans”, between distinct histones either within the same nucleosome or across nucleosomes, as well as between histones and DNA methylation [17]. In addition, different histone PTMs may cooperate in order to recruit specific complexes efficiently [18].

This review aims to provide a comprehensive summary of the ever-growing number of histone PTMs that have been studied over the last years. The molecular mechanisms involving histone PTMs, their genomic locations and implication in chromatin-based processes, as well as their interdependence will be discussed. Given the expanding dictionary of histone PTMs, in the following paragraphs I adopt the standard notation that has been conceived to represent them in a consistent and coherent format [19]. More particularly, the histone protein is first defined in uppercase letters, followed by a two-syllable word univocally indicating the individual modified residue and an abbreviation, in lowercase letters, conventionally indicating the type of modification.

## 2. Acetylation

In the early ‘60s, Phillips pioneered the field of histone PTMs originally reporting the N-acetylation of lysine residues in histones extracted from calf thymus [20]. Curiously, many authors attributed the achievement of this discovery to Allfrey and collaborators, although they clearly gave credit to Phillips in their first paper on this topic [21]. Undoubtedly, acetylation became the most studied histone PTM, so that nowadays we know that selected lysines are acetylated during specific biological processes, and that the overall histone acetylation level is dynamically modulated by the opposing action of two families of enzymes, namely the histone acetyltransferases (HATs) and the histone deacetylases (HDACs). This nomenclature, albeit currently used, is actually inappropriate because these enzymes can respectively transfer or remove a wide repertory of acyl species, besides the acetyl group, to both histone and non-histone target proteins (see below). In the case of acetylation, the HATs require acetyl-CoA as a cofactor to catalyse the transfer of an acetyl group to the ε-amino group of lysine side chains [22]. Based on their subcellular localization, HATs have been traditionally divided in two distinct types: type-A HATs are directly involved in histone acetylation in the context of nucleosomes, while type-B HATs acetylate free newly synthesized histones in the cytoplasm to facilitate their incorporation into the newly replicated chromatin [23,24]. HDAC enzymes reverse the activity of HATs, restoring the original unacetylated state of histone lysines. There are four classes of HDACs, whose members typically associate into multiprotein complexes showing rather low substrate specificity [25]. Collectively, both the mentioned enzyme families modify numerous, but not all, histone lysines in vivo [26]. Importantly, the HAT activity, differential substrate specificity and recruitment are modulated through multisubunit complex formation [27]. For example, yeast Gcn5 alone does acetylate free histones in vitro, but only when it is combined with the Ada2 and Ada3 subunits within the ADA and SAGA complexes, it can preferentially modify H2B enclosed into nucleosomes [28].

In principle, the observation that acetylation of lysine residues neutralizes their positive charge would suggest that HATs may reduce chromatin compaction by weakening the stabilizing influence of histone-DNA and inter-nucleosomal electrostatic interactions [29]. Accordingly, acetylation of H4K16 has been demonstrated to directly impact on chromatin structure by charge neutralization and loss of H4 tail flexibility, collectively reducing interaction with the neighbouring nucleosome [30,31,32]. In support of this evidence, ChIP-seq studies on a genome-wide scale revealed that site-specific acetylation is frequently associated with enhancers and promoters of actively transcribed genes [33,34,35,36]. However, there is some variation in the exact distribution of individual acetylation sites along the gene structure. For instance, H3K9ac peaks at the transcription start site, while H4K16ac shows a broader localization across gene bodies [36]. In sharp contrast, structural studies highlighted that lysine acetylation itself generally does not provoke major reorganization of DNA or core histones at the nucleosome level [37], leading to reasonably suppose that histone acetylation could generate recognition sites for effector proteins involved in the local regulation of chromatin organization and transcriptional activity.

## 3. Non-Acetyl Acylation

Besides acetylation, the ε-amino group of histone lysines can be alternatively modified by a growing repertoire of acyl moieties. These modifications deal with the transfer of at least four types of acyl groups: (1) short-chain hydrophobic groups, such as propionyl, butyryl, crotonyl, and the aromatic group benzoyl, proportionally increase the size and hydrophobicity of the lysine side chain compared to acetylation [38,39,40], (2) long-chain fatty acids, including hexanoyl, octanoyl, decanoyl, dodecanoyl, myristoyl, palmitoyl and stearoyl, have larger molecular volume and stronger hydrophobicity compared to the above mentioned short-chain groups, (3) polar groups, including β-hydroxy-butyryl and 2-hydroxy-isobutyryl, have branched hydrocarbon chains and provide a hydroxyl group implementing the hydrogen bond capability of the modified lysine [41,42], (4) negatively charged groups, including malonyl, succinyl, glutaryl, and lactoyl, switch the net electrostatic charge of the modified residue from +1 to −1 at a physiological pH [43,44,45].

All these acyl groups are derived from cellular metabolites and their acyl-CoA counterparts represent the source of diverse histone marks accompanying feedback and feedforward mechanisms of gene regulation and chromatin structure modulation. Notably, although the possible competition and functional interplay between distinct acylations on histone lysine residues require further investigation, these PTMs appear to be not redundant with each other and with acetylation.

For example, compared to histone acetylation, the transcriptional output associated with histone butyrylation is similar, while those associated with crotonylation and 2-hydroxy-isobutyrylation are greater and lesser, respectively [46,47,48]. 

The high reactivity of the various acyl-CoA types may lead to histones acylation independently of enzymatic involvement [49]. On the other hand, recent kinetic analysis confirmed promiscuous acylase/deacylase catalytic activities for the previously described HATs and HDACs, respectively [50]. Surprisingly, no enzyme showing specificity for only a given acylation has been discovered so far. Therefore, assuming that HATs interchangeably use different acyl-CoAs to modify histone lysine substrates, it could be reasoned that the relative abundance of the numerous acyl-CoA types would globally affect the differential histone acylation state, thereby connecting cell metabolism and gene regulation [51]. This hypothesis is supported by the fact that most of the available ChIP-seq datasets concordantly reveal the genomic co-occurrence of alternative acylation marks at distinctive proportional mixtures that correlate with specific functional outputs [46].

Site-specific acylation of core histones, such as H4K91glu, H3K122suc and H4K77suc, generally weakens histone-DNA interactions, thereby promoting nucleosome sliding [52,53,54]. Intriguingly, specific acylation patterns involving crotonylation, butyrylation and 2-hydroxy-isobutyrylation have been observed in the post-meiotic chromatin of murine spermatogenic lineage [39,46,55]. In addition, site-specific propionylation and butyrylation have been implicated in the expression of genes involved in lipid metabolism pathways in liver of fasted mice, while β-hydroxy-butyrylation was associated to activated transcription of genes involved in the starvation response [42,56]. Similarly, lysine benzoylation predominantly occurs on the N-terminal tails of H3 and H2B, specifically marking promoters of selected genes involved in phospholipase D signaling, glycerophospholipid metabolism, ovarian steroidogenesis and serotonergic synapse [40]. The unique physiological relevance of this epigenetic mark is revealed by the positive correlation between transcriptional upregulation of the mentioned genes and specific enrichment of benzoylation, but not other acylations, in their promoters. Moreover, while acetylated lysines are normally recognized by bromodomain-containing proteins, histone benzoyl-lysines preferentially recruit chromatin interactors bearing YEATS or DPF domains [57]. Among the known HDAC enzymes, only SIRT2 exhibited significant lysine debenzoylation activity in a catalytic screening, suggesting that it efficiently operates the reversal of benzoylation in vivo [40].

Lactylation of lysines strictly associates with the glycolysis pathway, relying upon the intracellular production of D-lactate in a dose-proportional fashion [58]. The HAT p300 has been implicated in the histone lactylation, and ChIP-seq analysis on samples derived from macrophages revealed that lysine lactylation specifically accumulates in the proximal promoter region of transcribed genes, being indicative of steady-state mRNA abundance [58,59]. Lysine lactylation can also occur via a non-enzymatic acyl transfer from S,D-lactoylglutathione, which is generated by the glyoxalase1 enzyme from the glycolytic by-product methylglyoxal [45,60]. 

In striking contrast to all these examples of positive correlation between histone acylation and gene expression, crotonylation at H3K9 has been associated to decreased transcription of growth genes during low-nutrient periods in *Saccharomyces cerevisiae*, most probably due to dynamic recruitment or repulsion of specific interactors not yet characterized [61].

## 4. Methylation

Unlike acetylation, histone methylation does not alter the net charge of modified residues and involves additional layers of complexity, because the side chains of two distinct amino acids can be modified at a different extent. More specifically, the ε-amino group of lysines can be mono-, di- or tri-methylated, while the ω-guanidine group of arginines can be mono- or di-methylated either symmetrically or asymmetrically [62,63].

Histone methyltransferases (HMTs) and demethylases (HDMs) enzymes carry out fine-tuning of the methylation state. Based on the specificity towards lysine or arginine, two major classes of HMTs can be distinguished, each of them containing a distinctive catalytic domain that transfers a methyl group from the S-adenosylmethionine donor. In contrast to other histone modifying enzymes, members of both classes are devoted to methylation of highly specific residues to an appropriate degree. For example, PRMT7 catalyses the formation of only mono-methyl-arginine, while PRMT5 electively determines symmetrical di-methylation of arginine [64,65]. Similarly, DIM5 univocally targets H3K9 for tri-methylation, while SET7/9 can only mono-methylate H3K4 in *Neurospora crassa* [66,67]. Notably, unlike acetylation, the different methylated histone sites may associate with diametrically opposed functional outcomes [68]. For instance, H3K4me3 and H3K36me3 have been primarily linked to a transcriptionally permissive chromatin environment [69], H3K4me1 is considered a predictive hallmark of active enhancers [70], while H3K27me3 and H4K20me3 have been generally associated with heterochromatin formation and gene silencing [71,72]. Interestingly, H3K9 provides an emblematic example of the dualistic association of methylation with repressed and active states of chromatin. Indeed, several studies concordantly showed that H3K9me3 represents a docking site for the α isoform of the heterochromatin protein 1 (HP1α), which assists the progress of chromatin condensation in various organisms [73,74]. Other studies came to apparently conflicting conclusions, demonstrating that H3K9me3 and the HP1γ isoform co-localize in mammalian euchromatic regions, where they are involved in transcriptional elongation and are rapidly lost upon cessation of transcription [75].

Analogously to other epigenetic mechanisms, the histone methylation status varies widely during adaptive responses to environmental influence. For example, H3K14me3 is not normally found in human chromatin but it is distinctively generated following infection of lung epithelial cells with the intracellular pathogen *Legionella pneumophila* [76].

As with the HMTs, HDM enzymes display a high grade of target specificity, being sensitive to the degree of methylation. For example, JMJD2A can reverse H3K9me3 to H3K9me2 but not to H3K9me1 [77]. Removal of methyl groups from mono-methylated and asymmetrically di-methylated arginines is most probably carried out by JMJD6 [78]. To date, this is the one and only enzyme possessing arginine demethylase activity directed toward histones, although this function has been questioned by contradictory evidence [79,80].

## 5. Citrullination

Indirect reversal of histone arginine methylation can be achieved through citrullination, which is the demethyl-imination modification of the primary monomethyl-imine group to a ketone group, yielding methylamine as a side-product. The conversion of methyl-arginine into citrulline is catalysed by the Ca^2+^-dependent peptidylarginine deiminase (PADI) enzymes [81,82]. In fact, PADIs exert a dual enzymatic activity, as they convert methylated arginine and unmodified arginine into citrulline via demethyl-imination and deamination, respectively [81,82]. Whatever is the case, PADI intervention determines the loss of the positive charge of the arginine side chains, which in turn promotes chromatin decondensation by affecting histone-DNA interactions [83]. In particular, citrullination of the single arginine residue at position 54 within the nucleosomal DNA binding region of H1 was found to lead to extensive chromatin decondensation [84]. Despite this, citrullination may be a repressive epigenetic mark on selected genes. For instance, in MCF-7 cells exposed to estradiol stimulation, level of histone citrulline on the estrogen-responsive *pS2* gene promoter was found to be inversely correlated to methyl-arginine level and *pS2* gene transcription [81,82]. Based on these findings it could be argued that the PADI4 enzyme not only controls the methylation status of multiple arginine residues in histone H3 and H4 by converting them into citrulline, but it also acts as a transcriptional co-repressor [81,82]. Given that the occurrence of an inverse demethyl-imination reaction catalysed by specific enzymes appears questionable, possibly histone replacement is required to regenerate unmodified arginine residues from citrulline.

## 6. Phosphorylation

Histone phosphorylation was first reported more than fifty years ago, yet this is among the more intensively investigated histone PTMs [85]. The overall level of phosphorylation in the chromatin depends upon the combined action of a plethora of kinases and phosphatases, which impose and remove the modification, respectively [86]. As usual, these enzymes are specifically recruited to their target sites on chromatin in the form of larger holoenzyme complexes, although for some kinases a direct binding activity has been described. This is the case of the mammalian MAPK1 kinase, which tethers to chromatin by means of an intrinsic DNA-binding domain [87]. 

Both the linker and core histones endure a large number of phosphorylation events on the hydroxyl group situated on the side chain of serine, threonine, and tyrosine residues [88]. The presence at these sites of a phosphate group, derived from the ATP donor, confers a heavy negative charge that undoubtedly induces relevant changes in the chromatin structure. A pertinent example is given by the phosphorylation of *Tetrahymena* histone H1 residues, which increases the dissociation rate of the linker histone from chromatin in vivo, through a so-called charge patch effect [89]. As a consequence, phosphorylation-dependent H1 ejection promotes chromatin relaxation, which in turn differentially affects the expression of some genes [89].

More generally speaking, phosphorylation of specific histone residues has been involved in several biological processes such as mitosis, meiosis, DNA damage response, apoptosis, and of course also gene expression [90,91,92]. For example, H3S10ph, which is the most thoroughly characterized histone phosphorylation site, is a highly dynamic modification showing an extremely rapid turnover during the cell cycle in plants, lower eukaryotes and mammal chromatin [93,94]. During mitosis and meiosis, this peculiar modification is deposited by the Aurora-B kinase and it is involved in chromosomal condensation by a so called phospho-methyl switch mechanism. In particular, the HP1 family proteins that normally bind to H3K9me3 show a significantly reduced affinity for H3 N-tails exhibiting the dual H3K9me3S10ph phospho-methylation mark, thus allowing the access of factors needed for proper condensation and segregation of the chromosomes [95,96]. In interphase, phosphorylation of H3S10 is instead mediated by two distinct kinases, namely Rak2 and Msk1, and it is associated with active transcription of numerous genes [97]. Contrarily, dephosphorylation of H3S10ph is carried out by the PP2A phosphatase and associates with transcriptional repression [98].

## 7. Ubiquitylation

Ubiquitylation involves only a single Ubiquitin moiety linked through an isopeptide bond between its C-terminal exposed glycine residue and the ε-amino group of specific target lysines within histones. Thus, mono-ubiquitylation peculiarly results in the addition of a relatively large polypeptide of about 8.5 kDa, rather than the small chemical groups described for many other histone PTMs. Unlike poly-ubiquitylation, which targets proteins to proteasome-dependent degradation, mono-ubiquitylation of histones is associated with the regulation of gene transcription and DNA damage response [99]. The site-specific attachment of Ubiquitin requires a sequential catalytic cascade consisting of activation, conjugation, and ligation, performed by the E1, E2, and E3 enzymes, respectively, while specialized isopeptidases attain the removal of Ubiquitin [99]. Comprehensive mapping of histone PTMs in the adult mouse brain revealed that ubiquitylation covers ~16% of total PTMs in core histones, localizing primarily on H2A and H2B [100]. The most extensively studied histone residues undergoing mono-ubiquitylation in higher eukaryotes are H2AK119 and H2BK120, both lying in the C-terminal tails of the two core histones [101]. More specifically, H2AK119Ub1 is generally associated with gene silencing and chromatin compaction, by stimulating deposition of the repressive mark H3K27me3 and stabilizing the association of linker histone H1 [102]. By contrast, H2BK120 is situated at the interface of adjacent nucleosomes, and its mono-ubiquitylation results in the inhibition of inter-nucleosome interactions to allow a more accessible conformation of the chromatin fiber [103]. Moreover, in the mammalian genome H2BK120Ub1 is largely confined within gene bodies, rather than promoters, where it directly stimulates histone methyltransferase activity of DOT1L and SET1 on H3K79 and H3K4, respectively, leading to a local enrichment of epigenetic marks closely correlated with the transcriptional elongation process [104,105,106,107].

Deciphering the intrinsic effects of ubiquitylation on the nucleosome structure and dynamics is considerably challenging due to the lack of a defined in vitro assay system. Nonetheless, it could be reasonably presumed that such a bulky modification is generally irreconcilable with the canonical nucleosome structure due to steric hindrances of the massive ubiquitin protein. For example, H2BK34 resides between two superhelical DNA gyres, and its mono-ubiquitilyation leads to drastic eviction of a H2A-H2B heterodimer from the nucleosome [108]. The resulting histone hexasome is quite stable and inefficiently incorporate a second, even unmodified, heterodimer, thus facilitating nucleosomal DNA breathing and accessibility [108]. Notably, a similar effect, although to a lesser extent, has been observed when ubiquitylation embraces histone residues residing at the nucleosome periphery, such as H2BK120 [108]. In addition, mono-ubiquitylation of the C-terminal H3K121, H3K122 and H3K125 residues has been reported to occur on histones that are not yet incorporated into yeast chromatin, outlining a ubiquitylation-dependent mechanism that control nucleosome assembly [109].

## 8. Sumoylation

Sumoylation involves the covalent attachment of either a single or multiple protein units among the small Ubiquitin-like modifier (SUMO) family members to histone lysines [110]. In principle, this modification could appear to be closely related and somehow redundant with respect to ubiquitylation, principally because Ubiquitin and SUMO proteins share comparable molecular size and three-dimensional structure, and both are managed by E1, E2, and E3 enzymes [111]. However, the two modifications are not related at all, at least not at an epigenetic level. Notably, sumoylation does not functionally replace ubiquitylation at H2BK120, strongly supporting the notion that the above-mentioned effects on gene expression cannot be explained merely by steric effects [112]. Sumoylation has been detected on all four nucleosomal histones at many genomic locations, where it has been involved in distinct functions. Mechanistically, histone sumoylation may inhibit chromatin compaction by affecting inter-nucleosomal interactions and by cross-regulating other histone PTMs. For example, sumoylated H2B and H3 in nucleosome occupying the yeast *gal1* gene were specifically associated with transcriptional repression and histone hypo-acetylation [113]. It has been suggested that transient sumoylation of H4 stimulates the local recruitment of repressive complexes bearing the HDAC1 deacylase and the H3K4-specific demethylase LSD1, allowing spatially restricted transcriptional repression by erasure of epigenetic marks that would otherwise lead to gene expression [114]. However, other studies reported that nucleosomes containing sumoylated H2B are the preferential binding platform for the ATP-dependent nucleosome remodeler RSC, which stimulates gene expression by altering the position, occupancy, and composition of nucleosomes [115].

Histone sumoylation may also play a relevant role in DNA repair, since persistent DNA double strand breaks incorporate nucleosomes containing sumoylated H2A.Z, which is absolutely required for DNA-lesion tethering to the nuclear periphery [116]. Furthermore, sumoylation of the yeast centromeric H3 variant Cse4 at K215 and K216 sites is required for normal kinetochore assembly and faithful chromosome segregation, while sumoylation at K65 prevents the aberrant spread of Cse4 into euchromatin [117,118].

## 9. Glycosylation

O-glycosylation, hereinafter referred to as glycosylation, relates to the addition of a single β-D-N-acetylglucosamine (GlcNAc) monosaccharide unit to the hydroxyl group of selected serine and threonine residues of the four core histones [119,120]. In contrast to most histone PTMs, there appear to be only two enzymes involved in the control of the glycosylation levels in chromatin, a transferase (OGT) that employs the donor substrate UDP-GlcNAc, and an antagonistic hydrolase (OGA) [121,122]. Intriguingly, OGA possesses an additional catalytic domain exhibiting HAT activity in vitro, suggesting that OGA would be able to simultaneously regulate the glycosylation and acetylation of histone residues [123,124]. On the other hand, OGA is not able to bind acetyl-CoA directly, suggesting that an ancillary partner would be required to ensure the putative HAT activity in vivo [124,125].

Histone glycosylation level normally fluctuates in response to variation in temperature and extracellular glucose abundance, as well as along cell cycle, decreasing during mitosis [120].

In most cases, glycosylation maps residues located in strategic positions of the nucleosome particle, including the loop 1 within the histone-fold domain, the lateral surface of the histone octamer stuck to the nucleosomal DNA, and the docking domain at the interface between the H2A/H2B dimer and H3/H4 tetramer, suggesting that this PTM could entail chromatin opening [119]. Structural effects induced by histone glycosylation has been investigated employing a site-selective chemoenzymatic assay, which allows installing GlcNAc onto mutagenically introduced cysteine residues on recombinant histones H2A and H2B, to generate semisynthetic assembled nucleosomes [126,127]. This approach confirmed that glycosylation of H2AT101, which lies at the dimer–tetramer interface of the nucleosome, indeed affects chromatin remodelling by destabilizing the H2A/H2B dimers and thus reducing nucleosome stability [128]. In the case of residues occupying nucleosome regions remote from any critical interface, rather than modulating nucleosome structure, histone glycosylation may favour the recruitment of protein complexes to mediate specific functional outputs. This is the case of H2BS112og, which impacts chromatin remodelling through direct recruitment of FACT, a pivotal histone chaperone complex, and the ubiquitin ligase BRE1A, which catalyses mono-ubiquitylation at H2BK120 [129,130].

Expectedly, genome-wide profiling of histone glycosylation by ChIP assays revealed that this modification is typically linked to transcriptional activation of selected genes [131]. Enrichment of O-glycosylation in these specific chromosomal sites likely depends on the stable association of OGT with prominent interactors, such as the Hcf1 transcriptional co-regulator, the Ten-eleven translocation proteins involved in the reversal of DNA methylation, and the Ubinuclein1 component of the histone chaperone HIRA complex [132,133,134].

## 10. ADP-Ribosylation

ADP-ribosylation is characterized by the synthesis of negatively charged monomers or polymers of ADP-ribose at specific acceptor amino acid residues of histone proteins [135]. The catalytic process requires nicotinamide adenine dinucleotide (NAD+) as donor substrate and is mediated by members of the ADP-ribosyltransferase family, while reversal of the modification is performed by specific hydrolases [136]. The number of studies regarding mono-ADP-ribosylation of histones remains very low at present. Only a small number of mono-ADP-ribosyltransferases, mainly ARTD-1, -3 and -10, have been reported to modify specific residues of linker and core histones in vertebrates [137,138,139]. Intriguingly, the SIRT-6 HDAC has been also implicated in histone mono-ADP-ribosylation by a dedicated catalytic domain [140]. This epigenetic modification occurs during DNA damage and repair, predominantly involving histone residues located on the nucleosomal surface [141]. Moreover, measurement of the histone ADP-ribosylation level in lysates derived from quiescent and dividing cells revealed a negative correlation between histone mono-ADP-ribosylation and cell proliferation [142]. Of note, poly-ADP-ribosylation exhibits a diametrically opposed trend, suggesting that the interconversion of mono- and poly-ADP-ribosylated histones may play a regulatory role in cell proliferation.

A few glutamate residues in H1 and H2B histones are the preferred acceptor sites for poly-ADP-ribosylation in native chromatin [143,144]. None of them, however, have been ultimately confirmed by mass spectrometry, probably due to the extremely reduced fraction of ADP-ribosylated histones, which is less than 1% of the total amount [145,146]. In principle, due to the bulky size and high negative electrostatic charge of poly-ADP-ribose, this PTM could diminish the local level of chromatin compaction. Indeed, high-resolution electron microscopy confirmed that poly-ADP-ribosylated chromatin reversibly adopts a more relaxed structure than its native counterpart, without dissociation of H1 and histone octamers from DNA [147,148]. Accordingly, specific enrichment in histone poly-ADP-ribosylation has been reported in transcriptionally active chromatin regions [149,150].

## 11. Biotinylation

Biotinylation is a reversible process relying on the covalent attachment of a B8 vitamin moiety to the ε-amino group of lysine residues of either linker or core histones [151,152]. Homeostasis of histone biotinylation is maintained by two biotinyl transferases, namely biotinidase (BTD) and holocarboxylase synthetase (HCS), which require biotinyl-ε-lysine and biotinyl-5′-AMP, respectively, as donor substrates [153,154]. Independent studies suggest that HCS plays a prominent role in histone biotinylation, while distinct enzymatic forms of BTD derived by alternative splicing seem to be involved in catalysing either biotinylation or debiotinylation of histones [155,156]. Globally, the human epigenome exhibits very low levels of histone biotinylation, in the order of attomoles of biotin incorporated into histones isolated from a million of cells [157,158]. However, the relative abundance of biotinylation marks depends on dietary biotin and may vary in response to cell proliferation [159,160]. Moreover, biotinylated histones are locally enriched at distinct repressed loci, including pericentromeric heterochromatin, telomeric repeats, and long tandem repeats [161,162,163,164]. In these chromatin regions, histone biotinylation generally co-localizes with the well-established repressive epigenetic mark H3K9me2, most probably as a consequence of the physical interaction between HCS and the H3K9-specific histone methyltransferase EHMT1 [165]. The involvement of histone biotinylation in gene repression has been corroborated by atomic force microscopy studies showing that H4K12bio and H4K16bio substantially facilitates chromatin condensation by increasing of almost 0.2 nucleosomal turns the length of DNA wrapped around the histone core octamer [166,167]. However, it should be emphasized that the authors of these studies employed recombinant histone H4 in which either K12 or K16 residues were mutated to cysteine for subsequent sulfhydryl-specific chemical reaction with maleimide-PEG-biotin to create biotinylated histones. Being this chemical modification quite different from the in vivo biotinylation, it somehow could have contributed to the observed changes on the nucleosome structure.

## 12. Monoaminylation

Recent evidence highlighted that transglutaminase enzymes can crosslink various monoamines to different protein targets, including histones, suggesting that neurotransmitters might be directly implicated in chromatin-associated processes. In particular, histone H3 but no other core histones can be modified by monoamines, namely serotonin and dopamine, in response to fluctuations in intracellular availability [168,169]. Transglutaminase-2 can covalently transfer both the mentioned monoamines to the carboxamide group of glutamine at position 5 on histone H3, yielding either serotonylated or dopaminylated nucleosomes. Transglutaminases can also remove monoamines from histones through deamidation reactions, indicating that monoaminylation is a reversible epigenetic modification [170,171].

While H3Q5 serotonylation plays a critical role for neurogenesis in mammals, dopaminylation on the same histone residue has been implicated in cocaine-induced transcriptional plasticity in dopamine neurons of the ventral tegmental area [169]. The H3Q5ser mark was found to coexist with adjacent H3K4me3 on the same histone tail at euchromatic regions of active gene expression in differentiating serotonergic neurons [168]. In this chromatin context, the TAF3 subunit of TFIID, normally involved in the recognition of H3K4me3, exhibits stronger binding preference for the H3K4me3Q5ser dual modification [172,173,174]. Concurrently, the H3K4me2/3 demethylases LSD1 and KDM5B/C are profoundly inhibited by the presence of the neighbouring H3Q5ser [174]. Most notably, deposition of the H3K4me3 mark is hampered upon disruption of the interaction between H3Q5ser and WDR5, which is a core subunit of the H3K4-specific MLL1 methyltransferase complex [174,175]. Taken together, these findings suggest that H3Q5ser is required to impose H3K4me3 and to stabilize it from dynamic turnover, enhancing its physical readout by downstream effectors.

## 13. Isomerization

Isomerization is the paradigm of non-covalent histone PTMs. It is the chemical conversion of an amino-acid residue into any of its isomeric forms, which possess the same chemical composition but exhibit different structure or configuration of atoms in space. In the case of proline, two possible isomeric conformations, either *cis* or *trans*, can be described depending on their dihedral angle referred to the peptide bond preceding the proline residue [176]. Although the two isomeric forms slowly switch one another in a spontaneous equilibrium state, enzymes belonging to the peptidyl-prolyl *cis-trans* isomerase (PPIase) superfamily accelerate such an interconversion process [177]. For example, in *S. cerevisiae* the PPIase Fpr4 specifically governs the *cis-trans* equilibrium of P38 on the histone H3 N-terminal tail [178]. In particular, the conformational status adopted by H3P38 disposes the histone tail either farther or closer to nucleosomal DNA, thereby affecting the opportunity for histone-DNA interaction and nucleosome stability, as well as the formation of higher-order chromatin structures [178].

Interestingly, Fpr4-dependent H3P38 isomerization regulates the methylation of the neighbouring H3K36 on the same histone tail. In particular, the *trans* conformation of H3P38 displaces the H3 tail far away from the nucleosome surface, creating favourable conditions for efficient recognition of H3K36 by the Set2 methyltransferase [178]. Reciprocally, in the presence of the *cis* H3P38 isomer, H3K36 no longer fits the active site of Set2 and it is more accessible for the demethylase JMJD2 [178]. The human ortholog of yeast Fpr4, hFKBP25, has been found to stably interact with HDAC-1 and -2, suggesting that an epigenetic crosstalk between lysine acetylation and proline isomerization could occur in vivo [179]. A confirmation for this hypothesis came from a more recent study showing that acetylation of H3K14 stimulates the *trans*-isomerization ratio of the adjoining H3P16, which in turn disfavours methylation of H3K4 [180].

Beyond the epigenetic control of chromatin plasticity at selected loci, histone proline isomerization can epigenetically define chromosome segregation fidelity in *S. pombe* [181]. In particular, a proline-rich heptapeptide motif of the N-terminal domain of the centromere-specific histone H3 variant CenPA is essential for ensuring precise chromosome segregation. Within this short amino acid stretch, *cis-trans* isomerization of P15 is carried out by the two FKBP-like PPIases encoded by the *SPBC1347.02* and *SPAC27F1.06c* loci, leading to accurate propagation of centromeric integrity in fission yeast.

Similar to proline, aspartic acid isomerization can occur spontaneously or can be catalysed by protein-L-isoaspartyl/D-aspartyl O-methyltransferase (PIMT) enzymes [182]. PIMTs catalyse the transfer of a methyl group from SAM to a carboxyl group of either an L-isoaspartyl or a D-aspartyl atypical residues. The methyl ester formed by this reaction is then converted into a succinimidyl residue, which is spontaneously hydrolyzed to produce a mixture of the physiologically dominant L-aspartate form and L-isoaspartate or occasionally D-aspartate [182]. Selective accumulation of L-isoaspartate has been observed at H2BD25, but not in other core histones, in chromatin derived from brain of PIMT knockout mice, indicating that rigorous control of H2BD25iso levels take place in neurons [183]. Intriguingly, in mouse brain a D-aspartate form of H2BD25 is present in approximately 12% of the overall H2B amount, and it is preferentially associated with active chromatin [184,185].

## 14. Glycation

Amongst the most prevalent non-enzymatic histone PTMs, glycation follows the Maillard reaction consisting in the condensation of reducing monosaccharides (especially glucose and ribose), their derivatives or glycolytic by-products with reactive nucleophile groups of amino acid residues, forming Schiff base adducts [186]. For example, both the amine functional groups of arginine and lysine side chains are susceptible to adduction by the glycolytic by-product methylglyoxal, resulting in N7-carboxymethyl-arginine and Nε-carboxymethyl-lysine, respectively [187]. The resulting adducts undergo rapid oxidization and rearrangement to form a plethora of more stable species generally referred to as advanced glycation end-products [188].

Recent reports revealed that methylglyoxal-mediated glycation sites mostly map on the lateral surface of the histone octamer, in close proximity to DNA, suggesting that they may lead to disruption of nucleosomal stability and global chromatin architecture by altering histone-DNA electrostatic interactions in a mechanism similar to acetylation [189,190]. Indeed, partial loss of secondary protein structure and incomplete nucleosome formation has been observed following glycation of both core and linker histones [191,192]. Accordingly, hyperglycemia or *glyoxalase1* gene knockout may allow accumulation of glycation adducts in mouse chromatin at the expense of canonical histone PTMs, leading to increased gene accessibility and global transcriptome alteration [189]. On the other hand, over-accumulation of adducts through high concentration or prolonged exposure to glycation agents results in robust cross-linking and hyper-compaction of the chromatin fiber, leading to the abrogation in DNA accessibility especially at transcription start sites [190].

Enzymatic reversion of Schiff base adducts is catalysed by the DJ-1 deglicase at lysine and arginine histone residues, while PADI4 shows deglycating activity solely towards arginines [190,193]. In this latter situation, PADI4-mediated citrullination not merely antagonizes histone glycation, but it shields citrullinated histones from undergoing glycation. Unfortunately, however, advanced glycation end-products cannot be reversed by both the mentioned enzymes.

## 15. Lipidation

As a result of oxidative stress in the cell, the fragmentation of polyunsaturated fatty acids yields numerous highly reactive α,β-unsaturated alkenals, including 4-hydroxy-2-nonenal (4-HNE) and 4-oxo-2-nonenal (4-ONE) [194,195]. Although sharing a very similar chemical backbone, the two mentioned species appear to arise independently, they do not interconvert metabolically and differ markedly in reactivity towards histones. In particular, 4-HNE primarily reacts with histidines via Michael addition, while 4-ONE forms ketoamide adducts via 1,2 addition to lysine residues [196,197]. A few 4-HNE-sensitive histidine residues have been identified exclusively in H2A, H2B and H4, lying in the globular and C-terminal domains of the histone proteins, and their adduction disrupts chromatin compaction by both 4-HNE steric bulk and neutralization of DNA-histone charge interactions [197,198]. Conceivably, these effects may make DNA more vulnerable to oxidative damage in Alzheimer disease [198,199]. All four core histones are instead modified by 4-ONE, being H2B the most heavily adducted species, and the adducted lysine and histidine residues mainly reside on the lateral surface of the nucleosome [196]. Unlike 4-HNE, adduction by 4-ONE elicits minor alterations in chromatin structure, although it can prevent de novo assembly of nucleosomes [196].

## 16. Formylation

In the last few years, formylation has been identified as a non-enzymatic histone PTM occurring on the ε-amino group of lysine residues under oxidative and nitrosative stress [200]. The formyl moiety predominantly derives from endogenous formaldehyde generated by oxidative demethylation of histones and nucleic acids, although it can occasionally arise from the 3′-formylphosphate intermediate in the 5′-oxidation of 2-deoxyribose in DNA [200,201]. In striking contrast to most of PTMs, the formyl-lysine adducts have been found to be rather equally distributed among different classes of histones from human lymphoblastoid cells TK6, suggesting that formylation is a fortuitous modification [202]. In spite of the chemical similarity between formyl and acetyl groups, formyl-lysines are not appreciably recognized by anti-acetyl-lysine antibodies, and they appear to be refractory to reversal by HDACs, with only the SIRT1 enzyme showing a minimum detectable activity value less than 10% in vitro [202,203].

Persistence of formyl-lysine adducts in individual histone proteins, coupled to the observation that they often mark residues that could be otherwise modified [204], strongly suggest that formylation can prevents the establishment of conventional histone PTMs. Additionally, formylation of residues involved in critical electrostatic interactions, such as H3K64, H4K77, H4K79, and H2BK34, may also impair organization and stability of the nucleosome particle [204].

## 17. Histone Tail Clipping

Numerous old and new studies reported that limited proteolysis of core and linker histone tails, referred to as histone tail clipping, is an evolutionarily conserved mechanism mediated by nuclear aminopeptidases and endopeptidases [205,206,207]. Although each study described distinct cleavage sites recognized by biochemically unrelated enzymes with clipping properties, these findings collectively suggest that the occurrence of histone tail clipping is crucial in many organisms and cell types for the regulation of various biological processes such as yeast sporulation and starvation, *Tetrahymena* conjugation, malaria parasite infection, mammalian embryonic stem cell differentiation, osteoclastogenesis, and senescence [208,209,210,211,212,213,214].

The occurrence of this irreversible epigenetic modification heavily impacts on chromatin plasticity and function. For instance, truncation of the N-terminal tails of core histones and/or the C-terminal tail of H2A destabilizes both intra- and inter-nucleosomal interactions, thereby increasing the breathability of chromatin [215,216,217,218]. Moreover, histone clipping could be thought as the most radical way to permanently reverse whatsoever PTM and to concomitantly abolish docking sites for chromatin-binding regulators mapping on histone tails. On the other hand, histone PTMs themselves might regulate the clipping incidence. For example, the JMJD5 and JMJD7 proteases specifically recognize and cleave at all possible degrees of arginine methylation on H2A, H3 and H4 histone tails through endopeptidase activity, progressively continuing to trim histone tails with aminopeptidase activity to generate tail-less nucleosomes [219]. Importantly, JMJD5 operates on nucleosomes at position +1, allowing RNA polII-dependent transcription elongation to occur smoothly [220]. In the case of histone H3, tail clipping can take place in the nucleosome or in the free pool of histones, preceding their deposition into specific genomic loci, which will integrate nucleosomes with unique properties both structurally and functionally [210]. An additional layer of complexity relies on the fact that the resected N-terminal H3 tail peptide may also directly bind the H3 mRNA, regulating its own translation [221].

A peculiar form of clipping without enzymatic engagement has been observed for the H2A C-terminal tail. In particular, Ni^2+^ ions are capable of forming a complex of octahedral geometry by binding the tetrapeptide sequence ESHH located at the C-terminal tail of histone H2A, through the carboxylate group on the side chain of glutamic acid and imidazole nitrogens on both of the histidine residues [222]. The particular steric arrangement of this square-planar complex is essential to assist Ni^2+^-dependent hydrolytic attack of the E121-S122 peptide bond, resulting in the removal of the C-terminal SHHKAKGK octapeptide from H2A [222,223,224]. Since the C-terminal tail normally interacts with histone H2B and linker DNA [225], the Ni^2+^-dependent truncation of H2A likely destabilizes the formation of higher-order structures of chromatin, thereby affecting gene transcription. Accordingly, several genes involved in neoplastic transformation exhibited either reduced or enhanced expression following H2A C-tail clipping, providing an epigenetic explanation for the mechanism of Ni^2+^-induced carcinogenesis [226].

Regardless of the enzymatic or non-enzymatic mechanism involved, as the cleaved tails cannot be re-ligated to the original histones, histone tail clipping can only be reversed through histone turnover or replacement.

## 18. Conclusions

At the present time, additional new types of histone PTMs have been identified, including asparagine deamidation [170,227], arginine and lysine carbonylation [228], tyrosine nitrosylation and hydroxylation [39,229], lysine 5′-hydroxylation [230], cysteine glutathionylation and sulfonylation [231,232], glutamine methylation [233], and H3K4me3 oxidation [234]. Intriguingly, although less well studied than those detailed in this review, most of them appear to be reversible and highly dynamic in a context-dependent fashion. However, the mechanisms by which these modifications can be imposed and reversed on the various classes of histones, as well as their structural and functional effects on chromatin are still largely unexplored.

There is a strong conceptual relationship between the combinatorial complexity of histone PTMs and the popular assumption of a “histone code”, whereby the sequential placement and/or removal of diverse histone PTMs on nucleosomes would univocally specify spatiotemporal patterns of gene expression [235]. From the perspective of this paradigm, histone PTMs are usually considered to be activating or repressive marks with respect to gene transcription, thus implying direct functional causality. Moreover, the histone-modifying enzymes are often referred to as writers and erasers, based on their respective capability of processing the forward or reverse modification reactions, while the so-called readers correspond to the protein complexes specifically recruited on chromatin by the various PTMs. Although the adoption of these metaphors became a commonplace in publications on this field, the mere existence of a histone code has been the subject of controversies and a conclusive one-to-one correspondence between histone PTMs and gene expression did not emerge [236]. In this regard, it should be emphasized that the alleged histone code lacks of the univocal information that should characterize each unit of a code. In fact, a same histone residue can potentially endure alternative types of PTM, and each of them may recruit multiple effector complexes in a context-dependent fashion, not to say that some enzymes can operate more than a single modification [237]. In addition, the two copies of each histone are not necessarily modified identically within a single nucleosome, giving rise to asymmetrically modified or bivalent nucleosomes, especially in the chromatin of stem cells or developing embryos [238,239]. Worth mentioning, the causal correlation between histone PTMs and downstream transcriptional outputs is formally questionable as well, since there are several cases in which a given histone PTM has found to be associated with antipodal effects on transcription activity. In this respect, it should be emphasized that nucleosomes are not static targets of modification enzymes within actively transcribed chromatin, which is instead characterized by a dynamic histone replacement process [240]. Finally, such a hypothetical code might not pertain specifically to histones, since proteins in general display complex patterns of covalent post-translational modifications.

Given all of that, probably it would be more appropriate to talk about a histone language, in which the different categories of PTM represent individual letters that will allow us to compose and/or decipher complex epigenetic words in the years to come.
